# Transformational and Transactional Leadership in the Polish Organizational Context: Validation of the Full and Short Forms of the Multifactor Leadership Questionnaire

**DOI:** 10.3389/fpsyg.2022.908594

**Published:** 2022-05-12

**Authors:** Beata Bajcar, Jolanta Babiak

**Affiliations:** Psychology and Ergonomics Group, Faculty of Management, Wrocław University of Science and Technology, Wrocław, Poland

**Keywords:** transformational-transactional leadership, MLQ, short form, employee's work outcomes, validation

## Abstract

The Multifactor Leadership Questionnaire (MLQ 5X Short) is the most frequently used leadership measure in scholarship and organizational practice. However, so far it has not been validated in the Polish context. Therefore, the present study aimed to validate and shorten the MLQ (5X Short) in the Polish organizational setting. A total sample of 1,065 employees (572 women and 493 men) from different organizations took part in two sessions of an online study. Respondents were between 18 and 70 years old (*M* = 40.1; *SD* = 12.9) with an average job tenure of 17.00 years (*SD* = 12.1). In subsample 1 (*n* = 539), using exploratory factor analysis, a three-factor structure of the MLQ full form (MLQ-FF) was established, comprising transformational-supportive, inspirational goal-oriented, and passive-avoidant leadership. Based on qualitative (i.e., content analysis) and quantitative criteria (psychometric parameters), we constructed an 18-item MLQ short form (MLQ-SF). Both forms were supported by the confirmatory factor analysis in subsample 2 (*n* = 526). The MLQ-FF and MLQ-SF factors displayed acceptable to high levels of item-related parameters (e.g., intra-class, inter-item, and item-total correlations), as well as scale-related reliability (e.g., internal consistency, temporal stability). Both forms indicated high convergent and predictive validity examined by correlations with authentic leadership and employee's work outcomes (i.e., work satisfaction, work effectiveness, work engagement, and organizational commitment) (subsample 3; *n* = 691). Our study provided the full and the short form of the MLQ as reliable and valid instruments, potentially suitable to measure leadership styles in academic research and organizational practice.

## Introduction

Transformational leadership theory, also referred to as full range leadership (FRL), has dominated the leadership field for over three decades (Avolio and Bass, 2004). The theory has shifted the long-standing scholarly focus from studying the mere economic goals of organizations to leadership behavior and human relations that play a central role in achieving long-term organizational outcomes (Bass, 1990). Within the scope of the FRL model, the Multifactor Leadership Questionnaire (MLQ) was developed to gauge leaders' behavior, ranging from transformational through transactional to laissez-faire, all of which are fundamental to leadership effectiveness (Bass and Bass, 2008). Transformational leadership pertains to the role modeling behaviors of a leader who seeks to transform her followers' attitudes and behaviors to perform beyond expectations. Transactional leadership is focused on reciprocal relationships of providing benefits for delivering performance. While transformational leadership is morale-uplifting, motivating, and inspiring, the transactional attitude exemplifies rather goal-oriented focus utilizing contingent rewarding. Laissez-faire leadership is usually considered as absence of leadership, and for the most part, is thought to negatively influence followers (Bass and Riggio, 2006).

The MLQ (5X Short), the most frequently used instrument in leadership research, includes nine dimensions of idealized influence attributed, idealized influence behavior, inspirational motivation, intellectual stimulation, individualized consideration, contingent reward, active management-by-exception, passive management-by-exception, and laissez-faire (Avolio and Bass, 2004). It measures five components of transformational leadership, three components of transactional leadership, and a laissez-faire style. The MLQ (5X Short), albeit not free from criticism (van Knippenberg and Sitkin, 2013; Batista-Foguet et al., 2021), has been primarily acknowledged as reflecting a full spectrum of transformational and transactional leadership styles (Antonakis et al., 2003). However, the structure of the MLQ (5X Short) and its previous versions, subjected to extensive factorial evaluations, has demonstrated inconsistent results across numerous studies that we comprehensively reviewed and presented in Table 1.

**Table 1:** Factor structure of the MLQ – review of studies.

**References**	**MLQ form**	**Participants**	**MLQ models tested**	**Results**	**Comments**
Bass (1985)	Form 1 73 items Rater form	USA; Senior executives *N* = 70 U.S. Army colonels *N* = 196	Exploratory factor analysis (EFA)	*6 factors*: 1.CH; 2.IS; 3.IC; 4.CR; 5.MBE; 6.Passive.	• Out of 143 items, 73 were extracted. • CH, IS, IC represented transformational CR, MBE - transactional leadership.
Hater and Bass (1988) Correlational study	Form 5 73 items Rater form	USA Delivery company *N* = 362	EFA	*6 factors:* 1.CH; 2.IC; 3.IS; 4.CR; 5.MBEA; 6.MBEP.	• MBE was split into active and passive dimensions.
Howell and Avolio (1993) Correlational study	Form 10 67 items Rater form	Canada Financial institution *N* = 322	EFA	*5 factors:* 1.Transformational (CH; IS; IC); 2.CR; 3.MBEA; 4.MBEP; 5.LF.	• EFA reduced the number of items to 31.
Yammarino et al. (1993) Longitudinal study	Form 1 44 of 73 items Rater form	USA US Navy Officers, *N* = 186 Subordinates of focal officers, *N* = 793	CFA: first-order factor model: 9-factors: CH; IC; IS; INSP; CP; CR; MBEA; MBEP; LF. EFA CFA: first-order factor model: 5-factors	*EFA: 5 factors*: 1.Transformational (CH, INSP, IS); 2.Transactional (CP, CR, IC); 3.MBEA; 4.MBEP; 5.LF. *CFA: 5 first-order factors*: 1.Transformational (CH, INSP); 2.Transactional (CP, CR, IC); 3.MBEA; 4.MBEP; 5.LF.	• To fit the military context the number of items was reduced to 44. • The 5-factor model included 27 items.
Druskat (1994) Correlational study	Form 8Y 40 items Rater form	USA Roman Catholic Church *N* = 6,359	EFA	*5 factors:* 1.CH, IC; 2.IS, INSP; 3.LF, MBEP; 4.CR; 5.MBEA.	• CH, IC, IS, INSP collapsed into 2 transformational factors. • MBEA and CR formed 2 transactional factors. • LF and MBEP formed one factor.
Tepper and Percy (1994) Validation study	Form X 24 of 73 items Rater form	USA S1*^*a*^*: Undergraduates/part-time and full-time employees *N* = 290 S2: Financial institutions managers *N* = 90	CFA S1*:* First-order factor model: (24 items): null, 1-, 2-, 8-factors; - (16 items): null, 1-, 2-, 4-, 5-factors. S2: Higher-order factor model (16 items): null, 1-, 2-, 3-factors	S1: *4 first-order factors:* 1.(CH, INSP); 2.CR; 3.IC; 4.IS. *5 first-order factors:* 1.CR; 2.CH; 3.INSP; 4.IC; 5.IS. S2: *2 higher-order factors:* 1.CH_I, CH_II, INSP_I, INSP_II; 2.CR.	• S1: the 24-item solutions were not acceptably fitted to the data. • The 4- and 5-factor models (16-items) were well fitted to the data. • More parsimonious 4-factor model was accepted. • S2: the 2- and 3-factor models were well fitted to the data. • More parsimonious 2-factor model was accepted.
Koh et al. (1995) Correlational study	Form 5S 73 items Rater form	Singapore Educational institution *N* = 844	EFA	*5 factors:* 1.CH, IC, IS; 2.CR; 3.MBEA; 4.MBEP; 5.LF.	• Seven factors emerged with eigenvalues above 1. • Due to interpretability a 5-factor model was accepted.
Bycio et al. (1995) Validation study	Form 1 40 of 73 items Rater form	Canada Registered nurses, health services, *N* = 1,376	CFA First-order factor models: null, 1-, 2-, 2- 5-factor	*5 first-order factors:* 1.CH; 2.IC; 3.IS; 4.CR; 5.MBE. *2 first-order factors:* 1.Active (CH, IC, IS, CR); 2.Passive (MBE).	• The 5-factor model was acceptably fitted to the data. • A simpler 2-factor of *active* vs. *passive leadership* solution was recommended.
Avolio et al. (1995)^b^ Validation study	Form 5X 36 items Rater form	USA Various companies *N* = 1,394	CFA First-order factor models: null, 1-, 2-, 3-, 6-, 7-, 8-, 8-, 9- factor.	*8 first-order factors:* 1.CH (IIA, IIB); 2.IM; 3.IS; 4.IC; 5.CR; 6.MBEA; 7.MBEP;8.LF. *9 first-order factors*: 1.IIA; 2.IIB; 3.IM; 4.IS; 5.IC; 6.CR; 7.MBEA; 8.MBEP; 9.LF.	• From among nine different models tested, the 8- and a 9-factor model were most adequately fitted to the data.
Den Hartog et al. (1997) Validation study	Form 8Y 40 items Rater form	Netherlands Various companies *N* = 1,200	EFA	*4 factors:* 1.Transformational (CH, INSP, IS, IC); 2.CR; 3.MBEA; 4.Passive (MBEP, LF). *3 factors:* 1.Inspirational (CH, INSP, IS, IC); 2.Rational-objective (CR, MBEA); 3.Passive (MBEP, LF).	• The 2-, 3-, 4- factor solutions were all well-interpretable.
Lievens et al. (1997) Correlational study	Form 8Y 40 items Rater form	Netherlands Various companies *N* = 319	EFA	*7 factors:* 1.(CH; INSP); 2.IS; 3.IC; 4.CR; 5.MBEA; 6.MBEP; 7.LF. *4 factors:* 1.Transformational (IS, IC, INSP); 2.CR; 3.MBEA; 4.Passive (MBEP, LF).	• Due to eigenvalue criteria, factor loadings, interpretability, and meaning, the 4-factor model was accepted. • The 4-factor solution included 30 items.
Geyer and Steyrer (1998) Correlational study	Form 5R 67 items Rater form	Austria Banks *N* = 376	CFA First-order factor model: 7-factor. EFA	*4 first-order factors*: 1.Core transformational (CH, IS, IM, IC^5^); 2.Individualized consideration (IC, CH); 3.Contingent reward (CR, IC); 4.Management -by-exception (MBE).	• The 4-factor model was revealed in EFA (67 items) and confirmed in CFA (35 items).
Avolio et al. (1999) Validation study	Form 5X 80 items Rater form	USA US and foreign companies, *N* = 3,786	CFA First-order factor models: null, 1-, 2-, 2-, 3-, 4-, 5-, 6-, 7- factor. Higher-order factor models: 2-, 3-, 3-factor.	*6 first-order factors:* 1.CH (IIA, IIB); IM; 2.IS; 3.IC; 4.CR; 5.MBEA; 6.MBEP, LF. *3 higher-order factors:* 1.Transformational (CH, IS), 2.Developmental /transactional (IC, CR), 3.Corrective-avoidant (MBEA, MBEP, LF).	• Fourteen independent samples were analyzed. • The number of items was reduced to 36.
Tejeda et al. (2001) Validation study	Form 5X 27 of 36 items Rater form	USA Various companies S1: *N* = 384, S2: *N* = 398, S3: *N* = 486, S4: *N* = 199	CFA First-order factor models: 9 factors (36 and 27 items) Higher-order factor model: 3-factor.	*9 first-order factors (27 items):* 1.AC; 2.II; 3.INSP; 4.IS; 5.IC; 6.CR; 7.MBEA; 8.MBEP; 9.LF. *3 higher-order factors (27 items):* 1.Transformational (AC, II, INSP, IS, IC); 2.Transactional (CR, MBEA, MBEP); 3.LF.	• Higher-order structure of the 27-item MLQ was supported in one sample only.
Vandenberghe et al. (2002) Validation study	Form 5X 50 items Rater form	Belgium Hospital nurses, *N* = 1059	CFA First-order factor models: null, 1-, 2-, 2-, 2-, 3-, 6-factor.	*6 first-order factors:* 1.MBEP; 2.MBEA; 3.CR; 4.Attributed CH; 5.IS; 6.IC. *1 higher-order factor* (Attributed CH, IS, IC, CR).	• Test for higher-order factor was restricted to transformational and CR, due to negative correlation between MBEA and MBEP (-0.41).
Bass et al. (2003) Correlational study	Form 5X 36 items Rater form	USA US Army *N* = 1,340 *N =* 1,335	CFA First-order factor models: 6-factor.	*6 first-order factors:* 1.CH (IIA, IIB); IM; 2.IS; 3.IC; 4.CR; 5.MBEA; 6.MBEP, LF.	• The 6-factor model was tested on 18 platoons and retested on a target sample of 72 platoons.
Antonakis et al. (2003) Validation study	Form 5X 36 items Rater form	USA Various companies S1: *N* = 3,368 S2: *N* = 6,525	CFA First-order factor models: 1-, 2-, 3-, 3-, 6-, 7-, 8-, 8-, 9-factor.	*3 first-order factors*: 1.Transformational; 2.Transactional; 3.Laissez-faire. *3 first-order factors:* 1.Transformational (IIA, IIB, IM, IS, IC); 2.Transactional (CR, MBEA); 3.Passive (MBEP, LF) *6 first-order factors:* 1.IIA, IIB, IM; 2.IS; 3.IC; 4.CR; 5.MBEA; 6.Passive (MBEP, LF). *7 first-order factors:* 1.IIA, IIB, IM; 2.IS; 3.IC; 4.CR; 5.MBEA; 6.MBEP; 7.LF. *8 first-order factors:* 1.IIA, IIB; 2.IM; 3.IS; 4.IC; 5.CR; 6.MBEA; 7.MBEP; 8.LF. *8 first-order factors:* 1.IIA; 2.IIB; 3.IM; 4.IS; 5.IC; 6.CR; 7.MBEA; 8.Passive (MBEP, LF). *9 first-order factors:* 1.IIA; 2.IIB; 3.IM; 4.IS; 5.IC; 6.CR; 7.MBEA; 8.MBEP; 9.LF.	• The 9-factor model was confirmed in S1 and S2, homogenous gender samples, in different contexts and samples: high-risk conditions, stable business conditions, majority males, majority females, lower-level leaders. • The 9-factor model has shown the best fit indices.
Rowold (2005) Validation study	Form 5X 36 items Rater form	Germany Government, managers, manufacturing, public transportation, students *N* = 1,267	CFA First-order factor models: null, 1-, 2-, 3-, 3-, 5-, 6-, 7-, 8-, 9-factor.	*9 first-order factors:* 1.IIA; 2.IIB; 3.IM; 4.IS; 5.IC; 6.CR; 7.MBEA; 8.MBEP; 9.LF. *3 first-order factors*: 1.Transformational (IIA, IIB, IM, IS); 2.Transactional (IC, CR, MBEA); 3.Passive (MBEP, LF). *5 first-order factors*: 1.Transformational (CH; IS; IC); 2.CR; 3.MBEA, 4.MBEP; 5.LF *6 first-order factors:* 1.CH (IIA, IIB); IM; 2.IS; 3.IC; 4.CR; 5.MBEA; 6.MBEP, LF. *7 first-order factors:* 1.CH; 2.IS; 3.IC; 4.CR; 5.MBEA; 6.MBEP; 7.LF. *8 first-order factors:* 1.IM; 2.II; 3.IS; 4.IC; 5.CR; 6.MBEA; 7.MBEP; 8.LF.	• All confirmed models displayed excellent fit indices.
Heinitz et al. (2005) Validation study	Form 5X 36 items Rater form	Germany Public administration S1: *N* = 1,311 S2: *N* = 879 S3: *N* = 650	CFA First-order factor models: 3-, 4-, 9-factor.	*3 first-order factors* (13 items): 1.Charismatic goal-orientation (IIB, IM, CR); 2.Passive-avoidant (MBEP, LF); 3.MBEA.	• In S1 and S2 the 9-factor model was not confirmed. In S2, 3-factor model was extracted. • In S3, due to eigenvalue and interpretability 4 factors (21 items) were not accepted, but • The 3-factor model (13 items) was confirmed.
Kanste et al. (2007) Validation study	Form 5X 36 items Rater form	Canada Nurses *N* = 601	EFA/ CFA First-order factor models: 1-, 2-, 2-, 3-, 6-, 7-, 8-factor.	*EFA: 3 factors (31 items):* 1.Rewarding transformational; 2.Passive laissez-faire; 3.Active management by exception *CFA: 6 first-order factors (25 items):* 1.IIA, IIB, IM; 2.IS; 3.IC; 4.CR; 5.MBEA; 6.MBEP, LF.	• EFA resulted in 3 factors including 31 items. • A modified, 25-item six-factor model was accepted.
Muenjohn and Armstrong (2008) Validation study	Form 5X 36 items Rater form	Australia Community sample, *N* = 138	CFA First-order factor models: 1-, 3-, 9-factor.	*9 first-order factors:* 1.IIA; 2.IIB; 3.IM; 4.IS; 5.IC; 6.CR; 7.MBEA; 8.MBEP; 9.LF.	• Due to small sample size CFA resulted in low fit indices of the tested models. • The 9-factor model was accepted.
Alonso et al. (2010) Validation study	Form 5X 36 items Rater form	Spain Various companies *N* = 954	CFA First-order factor models: 1-, 2-, 3-, 3-, 3-, 4-, 6-, 9-factor.	*4 first-order factors:* 1.Transformational (IIA, IIB, IM, IS); 2.Developmental/transactional (IC, CR), 3.Corrective (MBEA); 4.Passive/avoidant (MBEP, LF).	• Authors highlighted parsimony of the 4-factor model.
Edwards et al. (2012) Validation study	Form 5X 36 items Self/rater forms)	UK manufacturing companies *N* = 1,244	EFA/CFA First-order factor models: null, 1-, 2-, 2-, 3-, 3-, 3-, 4-, 5-, 6-, 7-, 9-factor.	*3 first-order factors*: 1.Active-constructive (IIA, IIB, IM, IS, IC, CR); 2.MBEA; 3.Passive-avoidant (MBEP, LF).	• None of the tested models fitted the data very well. • The 3-factor model was finally pursued in the analysis.
Boamah and Tremblay (2019) Validation study	MLQ 5X 32 items Rater form	Canada Registered nurses *N* = 378	EFA/CFA First-order factor model: 8-factor	*8 first-order factors:* 1.IIA; 2.IIB; 3.IM; 4.IS; 5 IC; 6.CR; 7.MBEA; 8.MBEP. *1 higher-order factor* (IIA, IIB, IM, IS, IC, CR, MBEP).	• LF scale was omitted. • The 8-factor model was verified in EFA and CFA. • Five factors described transformational, • Three factors represented transactional leadership.
**Factor analysis of MLQ's transformational leadership component**					
Densten and Sarros (1997) Validation study	Form 5R 67 items Rater form	Australia Law Enforcement Organization Senior police officers, *N* = 480	CFA Higher-order factor model: 6 factors EFA	*EFA: 11 first-order factors* *4 higher-order factors:* 1.II; 2.IM; 3.IC; 4.IS.	• In CFA, the 6-factor model (CH, IS, IC, CR, MBE, Passive) was not confirmed.
Hinkin et al. (1997) Correlational study	Form 5X 39-item Self/rater forms	USA Hotels General/middle managers, *N* = 123 Full-time employees, *N* = 158	EFA First-order factor models: - 4-factor (23 items) CFA- 3-factor (11 items).	*4 first-order factors:* 1.II; 2.IM; 3.IC; 4.IS. *3 first-order factors:* 1.IM; 2.IC; 3.IS.	• Four factors were yielded in EFA, but not confirmed in CFA • Finally, the 3-factor model was confirmed.
Tracey and Hinkin (1998) Correlational study	Form 5X 39 items Rater form	USA Hotels lower or middle-level managers *N* = 291	EFA/CFA First-order factor model: 4 factors	*CFA: 1 composite factor* (II, IM, IS, IC). *EFA: 5 factors:* 1.II, IC, IM; 2.IS; 3.II, IM; 4.IS; 5.IC, IS, IM.	• Four-factor model was not confirmed, but one-factor model (including 4 subscales) was confirmed. • In EFA, 5-factor model emerged for 39 items). • Finally, 5-factor solution (28 items) was accepted.
Carless (1998) Validation study	Form 5X 27 items Rater form	Bank Australia *N* = 1,389	CFA First-order factor models: 1-, 3-factor. Higher-order factor model: 1-factor	*3 first-order factors:* 1.Attributed CH; 2.IS; 3.IC. *1 higher-order factor* (Attributed CH, IS, IC)	• Transformational leadership was expressed as a single, higher-order construct.
Hemsworth et al. (2013) Correlational study	Form 5X 20 items Self form	Public sector chief executives, USA *N* = 372	CFA First-order factor model: 5-factor	*5 first-order factors*: 1.IIA; 2.IIB; 3.IM; 4.IS; 5.IC.	• Transformational leadership was represented by 20 items.

*IIA, Idealized influence attributed; IIB, Idealized influence behavior; IM, Inspirational motivation; IS, Intellectual stimulation; IC, Individualized consideration. CR, Contingent reward; MBEA, Active management-by-exception; MBEP, Passive management-by-exception; LF, Laissez-faire*.

The findings summarized in Table 1 demonstrate a significant scholarly effort to determine the high level of ecological validity of the MLQ (5X Short) and its previous forms. Some studies, conducted mainly in the North American context, attested the nine dimensions of the MLQ (5X Short) as best describing the theoretical FRL model. Other studies have uncovered different factorial solutions. Overall, they present a multidimensional nature of the transformational-transactional leadership and indicate that one universal model had not been conclusively agreed upon. The inconsistencies among studies' outcomes partly resulted from modifications to the MLQ (5X Short) and previous versions of the instrument. Some researchers merged scales and reassigned items between scales to achieve satisfactory fit indices and find the most suitable solution for their dataset. Attempts were also made to shorten the MLQ (5X Short) scale, and despite satisfactory psychometric properties, the abbreviated solutions were not further tested (Den Hartog et al., 1997; Heinitz et al., 2005; Kanste et al., 2007; Edwards et al., 2012).

Consequently, various factors were lost between studies, making it difficult to compare the results. Other sources of low consistency across studies were associated with the contextual characteristics of the research (Bycio et al., 1995; Den Hartog et al., 1997; Edwards et al., 2012). For instance, Antonakis et al. (2003) indicated industry, businesses, groups' heterogeneity vs. homogeneity, and the interactions between the leader and follower gender as potential contexts that influence the variations in the MLQ (5X Short) modeling. In sum, the MLQ (5X Short) received both support and criticism together with claims to refine the factors reflecting transformational and transactional constructs. It thus seems essential to evaluate the relevance and applicability of the MLQ model in a new national and organizational context, specifically to this study – the Polish organizations.

Noteworthy, the FRL model corresponds to other leadership constructs. In particular, the transformational component alludes to servant (Banks et al., 2018; Lee et al., 2020), ethical (Riggio et al., 2010), empowering (Lee et al., 2018), and authentic leadership styles (Avolio and Gardner, 2005; Lee et al., 2020). These concepts emphasize strong values, morals, ethics, and leaders' authentic attitudes toward their followers, organizations, and constituents (Banks et al., 2018). However, servant, ethical, and authentic leadership emphasize leader character rather than managerial competencies, whereas transformational-transactional leadership holds both qualities (Bass and Bass, 2008). Furthermore, in some instances, transformational leadership may be less authentic, but may never be inauthentic (Bass and Steidlmeier, 1999). Just as transformational-transactional leadership serves as the facilitator of positive work-related attitudes (Judge and Piccolo, 2004) so has authentic leadership been found to strongly relate to employees' job satisfaction, happiness, greater productivity, trust, and more positive working environment (Avolio and Gardner, 2005). Since authentic leadership contributes to open, truthful, and productive work atmosphere (Duarte et al., 2021), we assumed it holds great value for testing the convergent validity of the MLQ (5X Short) in the Polish context.

Thereto, empirical evidence has consistently demonstrated the significant role of the FRL model in predicting employee's outcomes, such as work performance across different types and criteria (Wang et al., 2011; Hetland et al., 2018; Steinmann et al., 2018; Lai et al., 2020; Ge et al., 2022), work satisfaction (Bass et al., 2003; Nohe and Hertel, 2017; Kammerhoff et al., 2019), trust in the leader (Breevaart and Zacher, 2019), work engagement (Tims et al., 2011; Miao et al., 2012), work motivation (Kanat-Maymon et al., 2020), and organizational commitment (Keegan and Hartog, 2004; Cho et al., 2019). The mechanisms explaining these relationships rely on assumptions that transformational-transactional leadership elicits general positive job attitudes of employees (Judge and Piccolo, 2004). In the same vein the exchange processes between leaders and employees are based on mutual trust which develops due to joint experiences (Nohe and Hertel, 2017; Breevaart and Zacher, 2019). In sum, transformational-transactional leadership is acknowledged as the wide-ranging leadership approach that facilitates conditions to create inclusive workplaces and positive outcomes for organizations (Bass and Riggio, 2006). These relationships serve as evidence for predictive validity of the MLQ (5X Short). It is thus our intention to uncover the predictive effectiveness of the FRL model in Polish organizations.

Although the MLQ (5X Short) is the most frequently used leadership measure in contemporary scholarship and practice, it has not been yet validated in the Polish organizational context. Hence, to use the measure in a new non-English-speaking environment we shall not rely on a mere translation but a soundly validated instrument. Furthermore, there is a scant number of studies that have undertaken to shorten the MLQ (5X Short) (e.g., Heinitz et al., 2005; Kanste et al., 2007). Parsimonious research instruments are currently highly valued due to often-imposed time constraints, like in large-scale research projects or hastening managerial practice. Therefore, considering this gap in the literature we aimed at validating and refining the MLQ (5X Short) in a population of Polish employees. Firstly, we assembled a Polish full version of the transformational-transactional leadership measure as the full form, called thereafter the MLQ-FF. We then examined its factorial structure and psychometric properties. Secondly, based on the qualitative (i.e., content analysis) and quantitative criteria (psychometric parameters) of the MLQ-FF we have developed a short version – the MLQ-SF. Next, we investigated the convergent and predictive validity of both forms.

In the light of the findings described above, we predicted significant relationships between the MLQ-FF and MLQ-SF and authentic leadership, as evidence of convergent validity. We hypothesized that transformational and transactional subscales would be positively associated with authentic leadership. The laissez-faire leadership would negatively relate to authentic leadership. In addition, predictive validity was established via the associations of the MLQ-FF and MLQ-SF with various employee work outcomes, like work engagement, organizational commitment, work effectiveness, and work satisfaction. We hypothesized positive associations between transformational and transactional leadership and work engagement, organizational commitment, work effectiveness, and work satisfaction. Lastly, we assumed that the laissez-faire leadership would negatively relate to work engagement, organizational commitment, work effectiveness, and work satisfaction.

## Materials and Methods

### Participants and Procedure

A total of 1087 employees from Polish organizations participated in the study. They represented various industries such as office and public administration (22%), services (17%), retail (19%), IT (14%), manufacturing (15%), and other (13%). To avoid the common method bias (Podsakoff et al., 2003), the study was conducted online in two sessions with a 4-week interval. Complete data were obtained from 1,065 participants (98% return rate; 572 women and 493 men) and included in the analyses. Respondents were between 18 and 70 years old (*M* = 40.1; *SD* = 12.9) with an average job tenure of 17.0 years (*SD* = 12.1). To ensure the external validity of the results and the generalization of conclusions for the study sample, the maximum number of participants in the target population was reached (Westland, 2012). In the first session, respondents completed MLQ (5X Short). The total sample was randomly split into two subsamples following the cross-validation framework to increase the measure's viability (de Rooij and Weeda, 2020). Subsample 1 included 539 individuals (294 women and 245 men), aged 19–70 years (*M* = 44.2; *SD* = 9.8), and an average tenure of 20.3 years (*SD* = 10.6). Subsample 2 consisted of 526 employees (278 women and 248 men) aged 18–70 years (*M* = 35.87; *SD* = 14.22), with an average tenure of 13.5 years (*SD* = 12.6). In the second session, 691 employees (subsample 3, 65% of a total sample) completed the remaining measures. Respondents (371 women; 320 men) were aged 19–70 years (*M* = 42.5, *SD* = 11.3) and a mean tenure was 19.1 years (*SD* = 11.2). Data from subsample 3 were used to examine the convergent and predictive validity of the MLQ.

All participants were informed about the anonymity of the survey and their participation was voluntary. All participants provided informed consent before inclusion in the study. Our study was carried out according to the ethical standards of the American Psychological Association and was approved by the university research ethics committee.

### Measures

#### Transformational-Transactional Leadership

The MLQ (5X Short; Rater Form) - a 36-item questionnaire, was used to measure transformational, transactional, and laissez-faire leadership styles (Avolio and Bass, 2004). It consists of nine dimensions: (1) idealized influence attributed (IIA), representing the attribution of charisma; (2) idealized influence behavior (IIB), reflecting the behavioral part of charisma; (3) inspirational motivation (IM), pertaining to the thought-provoking and motivating behavior of the leader; (4) intellectual stimulation (IS), expressing stimulating followers to unconventional and creative thinking; (5) individualized consideration (IC), demonstrating genuine interest in each follower's well-being and tending to their individual needs; (6) contingent reward (CR), representing fair and constructive management processes of rewarding good performance, both financially and psychologically; (7) active management-by-exception (MBEA), reflecting active monitoring of the follower work and taking corrective actions whenever necessary; (8) passive management-by-exception (MBEP), describing leader intervening behaviors upon occurrence of problems; and 9) laissez-faire (LF), expressing the absence of leadership or lack of involvement in leading (Avolio and Bass, 2004).

In this study, we used the Polish translation of the MLQ (5X Short) Rater Form provided by Mind Garden. To ensure conceptual equivalence to the English original version, the English and Polish versions of the questionnaire were verified by three independent expert judges from the psychology field. Then, all three versions were discussed, and differences were resolved. To gain higher confidence regarding item comprehension, five tenured employees appraised the level of difficulty in understanding of all items. Finally, 8 statements were slightly reformulated. After the results were verified, the final version of the Polish translation was agreed upon. The MLQ (5X Short) asked respondents to rate the leadership style of their respective leader using a 5-point Likert-type scale ranging from 0 (not at all) to 4 (frequently, if not always).

#### Authentic Leadership

A Polish validation of the Authentic Leadership Questionnaire (ALQ) was used to measure authentic leadership^1^ (Walumbwa et al., 2005; Wałachowska and Łaguna, 2018). The ALQ consists of four subscales: self-awareness (e.g., “My leader seeks feedback to improve interactions with others”), relational transparency (e.g., “My leader says exactly what he or she means”), internalized moral perspective (e.g., “My leader makes decisions based on his/her core beliefs.”), and balanced processing of information (e.g., “My leader listens carefully to different points of view before coming to conclusions.”). All items were rated on a 5-point Likert-type scale ranging from 1 (strongly disagree) to 5 (strongly agree). In this study, Cronbach's α for the subscales ranged between 0.75 and 0.91, and the total score of the authentic leadership scale was 0.86.

#### Work Engagement

Work engagement was measured using nine items drawn from the Polish version of the Utrecht Work Engagement Scale (UWES-SF; Schaufeli et al., 2006; Szabowska-Walaszczyk et al., 2011). The scale consists of three subscales with three items each: vigor (e.g., “When I get up in the morning, I feel like going to work.”), dedication (e.g., “I am enthusiastic about my job.”), and absorption (e.g., “When I am working, I forget everything else around me.”). A higher total score indicates a higher level of work engagement. Cronbach's α for the subscales ranged between 0.81 and 0.88, and the total score of work engagement was 0.93.

#### Organizational Commitment

To measure organizational commitment, the Organizational Commitment Scale (Meyer and Allen, 1991), validated in Poland (Bańka et al., 2002), was used. It included three subscales: affective commitment (e.g., “The organization has a great deal of personal meaning for me.”), normative commitment (e.g., “This company deserves my loyalty.”), and continuance of commitment (e.g., “It would be very hard for me to leave my company, even if I wanted to do so.”). Responses were rated on a 5-point Likert-type scale ranging from 1 (strongly disagree) to 5 (strongly agree). Cronbach's α for the subscales ranged between 0.73 and 0.86, and for the total score it was 0.91.

#### Work Effectiveness

Employee's work effectiveness was assessed with one item, “How would you rate your work effectiveness?” on a 5-point Likert-type scale ranging from 1 (very low) to 5 (very high).

#### Work Satisfaction

We assessed employee's work satisfaction with one item, “How satisfied are you with your work?” on a 5-point Likert-type scale ranging from 1 (completely unsatisfied) to 5 (completely satisfied).

### Analyses

A confirmatory factor analysis (CFA) was conducted to test the various factor solutions of the MLQ (5X Short) established in earlier studies. Next, using an exploratory factor analysis (EFA), we identified the MLQ factor structure specific to the Polish organizational context (in subsample 1), which was then verified by CFA (in subsample 2). As suggested by Byrne (2016), root mean square error of approximation (RMSEA) and standardized root mean square residual (SRMR) values below 0.08 indicate an acceptable fit, and values below 0.05 indicate a very good fit. The adjusted goodness of fit index (AGFI), comparative fit index (CFI), and Tucker-Lewis fit index (TLI) values higher than 0.90 show a good model fit. Based on the full form of the MLQ (MLQ-FF) factor solution, the short form (MLQ-SF) was developed by conducting an EFA (in subsample 1) and a CFA (in subsample 2). Next, psychometric characteristics of both forms of the MLQ were identified. To examine the measure's scale-level (internal consistency, temporal stability) and item-level reliability indices (item-total, inter-class, and intra-class correlations) were assessed separately in subsamples 1 and 2. In addition, the tests of the differences between women and men in relation to the MLQ dimensions were conducted. Due to not normally distributed data, we used a nonparametric Mann-Whitney *U*-test. The convergent and predictive validity were verified by correlating both forms of the MLQ, authentic leadership and various work outcomes (in subsample 3). As a follow-up study, the temporal stability of the MLQ-FF and MLQ-SF was examined. Analyses were performed using the statistical package IBM SPSS 25.0, and the CFA was performed using AMOS 25.0 software.

## Results

### Confirmation of the Previous Factor Structures of the MLQ (5X Short) in a Total Sample

First, we tested various factor solutions of the MLQ (5X Short) established in earlier studies (see Table 2).

**Table 2:** The MLQ factor models from previous studies tested in the current study.

**Factor model**	**Authors**	**Factor**								
		**1**	**2**	**3**	**4**	**5**	**6**	**7**	**8**	**9**
2-factor model v1	Avolio et al. (1999)	IIA, IIB, IM, IS, IC, CR	MBEA, MBEP, LF							
2-factor model v2	Antonakis et al. (2003)	IIA, IIB, IM, IS, IC, CR, MBEA	MBEP, LF							
3-factor model v1	Kanste et al. (2007); Edwards et al. (2012)	IIA, IIB, IM, IS, IC, CR	MBEA	MBEP, LF						
3-factor model v2	Den Hartog et al. (1997); Antonakis et al. (2003)	IIA, IIB, IM, IS, IC	CR, MBEA	MBEP, LF						
3-factor model v3	Tejeda et al. (2001)	IIA, IIB, IM, IS, IC	CR, MBEA, MBEP	LF						
3 factor model v4	Rowold (2005)	IIA, IIB, IM, IS	IC, CR, MBEA	MBEP, LF						
4-factor model v1	Alonso et al. (2010)	IIA, IIB, IM, IS	IC, CR	MBEA	MBEP, LF					
4-factor model v2	Den Hartog et al. (1997)	IIA, IIB, IM, IS, IC	CR	MBEA	MBEP, LF					
5-factor model	Koh et al. (1995); Rowold (2005)	IIA, IIB, IM, IS, IC	CR	MBEA	MBEP	LF				
6-factor model	Avolio et al. (1999); Antonakis et al. (2003); Rowold (2005)	IIA, IIB, IM	IS	IC	CR	MBEA	MBEP, LF			
7-factor model	Avolio et al. (1999); Rowold (2005)	IIA, IIB, IM	IS	IC	CR	MBEA	MBEP	LF		
8-factor model v1	Rowold (2005)	IIA, IIB	IM	IS	IC	CR	MBEA	MBEP	LF	
8-factor model v2	Antonakis et al. (2003)	IIA	IIB	IM	IS	IC	CR	MBEA	MBEP, LF	
9-factor model	Antonakis et al. (2003)	IIA	IIB	IM	IS	IC	CR	MBEA	MBEP	LF

*IIA, Idealized influence attributed; IIB, Idealized influence behavior; IM, Inspirational motivation; IS, Intellectual stimulation; IC, Individualized consideration; CR, Contingent reward; MBEA, Active management-by-exception; MBEP, Passive management-by-exception; LF, Laissez-faire*.

The series of conducted CFA indicated that the 9-factor original model and all other tested models resulted in unsatisfactory fit indices (Supplementary Table 1 in the Supplementary Material).

### Factor Structure and Reliability of the MLQ-FF and the MLQ-SF – Subsample 1

Given that the previous factor structures of the MLQ (5X Short) were not confirmed in the Polish sample, we were prompted to search for new solutions. We thus performed an EFA in subsample 1 (*n* = 539). Following Kline's (2016) recommendation and to test the suitability of the data for factor analyses we calculated the overall Kaiser-Meyer-Olkin measure and Bartlett's test of sphericity, which were significant (KMO =0.96; χ2(630) = 13,920.35, *p* < 0.001). Based on the principal component method, scree plot criterion, and Promax rotation, a two- and three-factor solution was extracted. Items with factor loadings higher than 0.40 were considered for further analysis.

In the two-factor model, all items that represented transformational and transactional leadership loaded on the first factor and explained 41% of the variance. The second factor included 10 items of the passive and avoidant leadership. These two factors accounted for 54% of the total variance. In the three-factor solution (see Table 3), the first factor (13 items) contained all IC items, three items from IA and IS, one item from IB, two items from CR, and it explained 42% of the variance. The core of the first factor was transformational leadership, which emphasized individual support and recognition, so we called it transformational-supportive leadership. The second factor of 14 items combined IM (all items), MBEA (all items), IB (three items), CR (two items), and IS (one item) and explained 12% of the variance. The content of the items implied a leader's inspirational attitude with a strong tendency to complete tasks and accomplish goals. Hence, it is referred to as inspirational goal-oriented leadership. The third factor explained 4% of the variance, included 8 items from the MBEP and LF scales and expressed passive-avoidant leadership. Due to the cross-loading of item 25, we decided to exclude it from further analyses.

**Table 3:** Means, standard deviations, factor loadings, and item-total correlations for MLQ-FF and MLQ-SF in subsample 1.

**Item**	**MLQ (5X Short) subscales**	**MLQ-FF**				**MLQ-SF**	
		***M***	***SD***	**FL**	***r*_**item−total**_**	**FL**	***r*_**item−total**_**
Factor 1: Transformational-supportive							
Item 19	IC	2.92	1.11	0.86	0.72	0.97	0.70
Item 18	IIA	2.67	1.17	0.83	0.78	0.90	0.76
Item 31	IC	2.80	1.15	0.82	0.87	0.88	0.86
Item 29	IC	2.69	1.04	0.80	0.50		
Item 30	IS	2.91	1.07	0.71	0.81	0.71	0.81
Item 21	IIA	3.17	1.10	0.71	0.81	0.82	0.81
Item 1	CR	3.13	1.17	0.70	0.82	0.84	0.80
Item 10	IIA	2.77	1.17	0.68	0.82		
Item 32	IS	2.87	1.06	0.63	0.81	0.66	0.81
Item 23	IIB	2.98	1.05	0.60	0.76		
Item 2	IS	3.01	1.06	0.60	0.79		
Item 15	IC	2.81	1.12	0.56	0.75		
Item 35	CR	3.14	1.02	0.52	0.74	0.62	0.75
Factor 2: Inspirational goal-oriented							
Item 13	IM	3.28	0.98	0.80	0.76	0.85	0.73
Item 14	IIB	3.18	1.04	0.74	0.77	0.80	0.73
Item 27	MBEA	2.82	0.91	0.67	0.48	0.82	0.47
Item 4	MBEA	3.03	1.02	0.61	0.14		
Item 26	IM	3.00	1.02	0.61	0.72	0.67	0.71
Item 24	MBEA	3.08	0.98	0.57	0.64	0.59	0.60
Item 34	IIB	3.00	1.04	0.56	0.70	0.62	0.69
Item 36	IM	3.54	0.87	0.56	0.48		
Item 11	CR	3.06	1.06	0.52	0.77		
Item 6	IIB	3.11	1.05	0.51	0.60		
Item 9	IM	3.28	1.00	0.49	0.69		
Item 22	MBEA	2.88	0.98	0.44	0.59		
Item 16^a^	CR	3.01	1.07	0.44	0.74		
Item 8	IS	3.23	0.97	0.41	0.67		
Factor 3: Passive-avoidant							
Item 20	MBEP	2.57	0.64	0.85	0.65	0.88	0.63
Item 12	MBEP	2.67	0.69	0.79	0.65	0.81	0.62
Item 28	LF	2.48	0.68	0.77	0.63	0.75	0.60
Item 5	LF	2.64	0.67	0.76	0.68	0.75	0.63
Item 33	LF	2.70	0.26	0.72	0.65		
Item 7	LF	2.67	0.50	0.70	0.65		
Item 17^b^	MBEP	2.82	1.03	0.52	0.28		
Item 25^c^	IIA	2.89	1.19	0.52*	-		
Item 3	MBEP	3.23	1.08	0.45	0.35		

*n = 539. FL – factor loadings. r _item−total_ – item-total correlation_._ MLQ-FF: full form. MLQ-SF: short form*.

a *Item 16 loaded onto factor 1 with factor loading of 0.44*.

b *Item 17 loaded onto factor 1 with a factor loading of 0.51*.

c *Item 25 loaded onto factors 1 and 2 with factor loadings of −0.48 and 0.44, respectively. IIA – Idealized influence attributed*.

*IIB, Idealized influence behavior; IM, Inspirational motivation; IS, Intellectual stimulation; IC, Individualized consideration*.

*CR, Contingent reward; MBEA, Active management-by-exception; MBEP, Passive management-by-exception; LF, Laissez-faire*.

In total, the three factors explained 58% of the variance. All factor loadings ranged between 0.41 and 0.86. Complete results for the three-factor solution for the MLQ-FF are presented in Supplementary Table 2 in the Supplementary Material.

In the next step, we attempted to shorten the obtained MLQ-FF. Considering both quantitative (i.e., psychometric parameters) and qualitative criteria (i.e., item-content analysis), we have selected items with the highest factor-loadings and item-total correlations that simultaneously represented the content of the nine original MLQ (5X Short) subscales. Two items per each of the 9 subscales were selected, creating an 18-item form– the MLQ-SF. The shortened version was subjected to an EFA in subsample 1, and a three-factor solution with eigenvalues above 1 was extracted, which explained 67% of the total variance (Factor 1: 47%, Factor 2: 14%, Factor 3: 6%, respectively). All factor loadings for the MLQ-SF items ranged from 0.50 to 0.82 (see Table 3).

The subsequent analyses were performed on the MLQ-FF and MLQ-SF simultaneously hence, we present these results together. The transformational-supportive and inspirational goal-oriented factors of the MLQ-FF and MLQ–SF were highly intercorrelated (0.84 and 0.77, respectively), and both correlated negatively and slightly to moderately with the passive-avoidant factor (from−0.11 to−0.26). According to Cohen's (1988) interpretation of the magnitude of correlation, correlations in the order of 0.10 are “small,” those of 0.30 are “medium,” and those of 0.50 are “large.” The internal consistency of the MLQ-FF subscales was between 0.84 and 0.96 and of the MLQ-SF between 0.80 and 94. The intraclass correlation (ICC) values ranged between 0.84 and 0.95 for the MLQ-FF and between 0.80 and 0.94 for the MLQ-SF. The item-total correlations ranged from 0.14 to 0.87 for the MLQ-FF and from 0.47 to 0.86 for the MLQ-SF. The inter-item mean correlation ranged from 0.39 to 0.62 for the MLQ-FF and from 0.51 to 0.66 for the MLQ-SF (all inter-item correlations ranged from 0.01 to 0.82 for the MLQ-FF and from 0.34 to 0.79 for the MLQ-SF). All item- and scale-related parameters are presented in Supplementary Table 4 in the Supplementary Material.

Next, a Mann-Whitney U test revealed a significant difference between women and men in passive-avoidant leadership for MLQ-FF (*U* = 31633.50*, z* = −2.44, *p* = 0.015) and for MLQ-SF (*U* = 32,375.00, *z* = −2.03, *p* = 0.042). Men perceived their leaders as displaying passive leadership to a higher extent (MLQ-FF: *M* = 22.35, *SD* = 5.52, *Mdn* = 22.00; MLQ-SF: *M* = 10.62, *SD* = 3.19, *Mdn* = 11.00) than women did (MLQ-FF: *M* = 21.32, *SD* = 5.97, *Mdn* = 21.00; MLQ-SF*: M* = 10.14, *SD* = 3.41, *Mdn* = 10.00). However, there were no significant differences in transformational-supportive and inspirational goal-oriented factors for both MLQ forms (see Supplementary Table 5 in the Supplementary Material). Additionally, the MLQ-FF and MLQ-SF factors did not correlate significantly with age (see Table 4).

**Table 4:** Means, standard deviations, Cronbach α, and correlations for all analyzed variables.

**Variables**	**MLQ-FF**						**MLQ-SF**		
	***M***	***SD***	**Cronbach α**	**1**	**2**	**3**	**4**	**5**	**6**
*MLQ-FF subscales (subsample 1)*									
1. Transformational-supportive^a^	37.87	11.52	0.96		.				
2. Inspirational goal-oriented^a^	43.48	9.61	0.91	0.84^**^					
3. Passive-avoidant^a^	21.79	5.79	0.84	−0.25^**^	−0.11^*^				
*MLQ-SF subscales (subsample 1)*									
4. Transformational-supportive^a^	23.61	7.43	0.94	0.99^**^	0.82^**^	−0.27^**^			
5. Inspirational goal-oriented^a^	18.36	4.60	0.86	0.77^**^	0.95^**^	−0.08	0.75^**^		
6. Passive-avoidant^a^	10.36	3.32	0.80	−0.26^**^	−0.14^**^	0.95^**^	−0.28^**^	−0.12^**^	
7. Age	44.23	9.81	-	0.01	0.01	−0.03	0.01	−0.01	−0.05
*MLQ-FF subscales (subsample 2)*									
1. Transformational-supportive^b^	38.52	10.88	0.93		.				
2. Inspirational goal-oriented^b^	44.02	9.90	0.90	0.83^**^					
3. Passive-avoidant^b^	19.96	5.98	0.83	−0.30^**^	−0.16^**^				
*MLQ-SF subscales (subsample 2)*									
4. Transformational-supportive^b^	24.20	7.13	0.90	0.98^**^	0.82^**^	−0.33^**^			
5. Inspirational goal-oriented^b^	18.74	4.67	0.82	0.79^**^	0.95^**^	−0.13^**^	0.77^**^		
6. Passive-avoidant^b^	9.34	3.61	0.83	−0.31^**^	−0.20^**^	0.94^**^	−0.34^**^	−0.17^**^	
7. Age	35.87	14.22	-	−0.11^*^	−0.11^*^	0.14^**^	−0.13^**^	−0.10^*^	0.16^**^
*ALQ subscales*									
8. Self-awareness^c^	12.18	3.90	0.91	0.78^**^	0.70^**^	−0.29^**^	0.77^**^	0.64^**^	−0.30^**^
9. Relational transparency^c^	15.42	4.24	0.81	0.70^**^	0.66^**^	−0.28^**^	0.70^**^	0.61^**^	−0.29^**^
10. Internalized moral^c^	12.64	4.01	0.75	0.67^**^	0.62^**^	−0.30^**^	0.66^**^	0.58^**^	−0.33^**^
11. Balanced processing of information^c^	9.26	2.93	0.86	0.74^**^	0.66^**^	−0.31^**^	0.74^**^	0.60^**^	−0.33^**^
12. Authentic leadership (total)^c^	49.04	13.85	0.86	0.80^**^	0.73^**^	−0.32^**^	0.79^**^	0.67^**^	−0.34^**^
*Employee outcomes*									
13. Vigor^c^	11.14	2.76	0.88	0.41^**^	0.33^**^	−0.09^*^	0.41^**^	0.33^**^	−0.07
14. Dedication^c^	11.90	2.96	0.87	0.41^**^	0.35^**^	−0.09^*^	0.40^**^	0.34^**^	−0.10^*^
15. Absorption^c^	11.47	2.81	0.81	0.37^**^	0.33^**^	−0.06	0.36^**^	0.32^**^	−0.07
16. Work engagement (total)^c^	34.50	7,85	0.93	0.43^**^	0.37^**^	−0.09^*^	0.42^**^	0.36^**^	−0.08^*^
17. Affective commitment^c^	18.90	5.23	0.86	0.60^**^	0.48^**^	−0.14^**^	0.59^**^	0.43^**^	−0.14^**^
18. Normative commitment^c^	17.59	5.28	0.86	0.46^**^	0.36^**^	0.01	0.44^**^	0.35^**^	0.02
19. Continuance commitment^c^	18.36	4.49	0.73	0.09^*^	0.08^*^	0.15^**^	0.08^*^	0.08^*^	0.16^**^
20. Organizational commitment (total)^c^	54.86	12.87	0.91	0.46^**^	0.37^**^	0.01	0.45^**^	0.35^**^	0.01
21. Work effectiveness^c^	3.92	0.77	-	0.30^**^	0.25^**^	−0.17^**^	0.30^**^	0.23^**^	−0.16^**^
22. Work satisfaction^c^	3.97	0.99	-	0.33^**^	0.28^**^	−0.13^**^	0.34^**^	0.26^**^	−0.13^**^

a *n = 539*.

b *n = 526*.

c *n = 691*.

* *p < 0.05*,

** *p < 0.01. MLQ-FF, full form; MLQ-SF, short form*.

### Factor Structure and Reliability of the MLQ-FF and MLQ-SF – Subsample 2

The three-factor solution of the MLQ-FF extracted in subsample 1 was tested by CFA in subsample 2. Kaiser-Meyer-Olkin measure and the Bartlett's test of sphericity (KMO =0.95, χ2(630) = 10,357.48, *p* < 0.001) were statistically significant, indicating good suitability of the data for factor analysis. The model fit indices exceeded the recommended cut-off criteria (Byrne, 2016), χ^2^ = 743.48, df = 477, χ^2^/df = 1.56, *p* < 0.001, RMSEA = 0.033, GFI = 0.93, AGFI = 0.90, CFI = 0.97, TLI =0.97, SRMR =0.052. All standardized factor loadings ranged from 0.14 to 0.83 (see Supplementary Table 3 in the Supplementary Material). The CFA conducted on the MLQ-SF also indicated good fit of the factor model to the data (χ^2^ = 188.30, df = 111, χ^2^/df = 1.70, *p* < 0.001, RMSEA = 0.036, GFI = 0.96, AGFI = 0.94, CFI = 0.98, TLI = 0.98, SRMR = 0.041). All standardized factor loadings ranged from 0.54 to 0.82 (see Supplementary Table 3 in the Supplementary Material).

In subsample 2, the transformational-supportive and inspirational goal-oriented factors of both MLQ forms were highly intercorrelated, and they correlated negatively at a low to moderate level with the passive-avoidant factor. Internal consistency of the MLQ-FF subscales was between 0.83 and 0.93 and between 0.82 and 0.90 for the MLQ-SF. The intraclass correlation (ICC) values ranged between 0.83 and 0.93 for the MLQ-FF and between 0.82 and 0.90 for the MLQ-SF. The item-total correlations were above 0.19 for the MLQ-FF and above 0.48 for the MLQ-SF, the inter-item mean correlation was between 0.38 to 0.49 for the MLQ-FF and between 0.43 and 0.55 for the MLQ-SF (all inter-item correlations ranged from 0.05 to 0.76 for the MLQ-FF and from 0.28 to 0.76 for the MLQ-SF).

In addition, a Mann Whitney test revealed significant differences between women and men in the transformational-supportive leadership in both MLQ forms (MLQ-FF: *U* = 30376.50, *z* = −2.35, *p* =*0.0*19, MLQ-SF: *U* = 30563.00, *z* = −2.25, *p* = *0.0*25). Men's rating of their leaders' transformational-supportive behavior was higher (MLQ-FF: *M* = 39.65, *SD* = 10.22, *Mdn* = 41.00; MLQ-SF: *M* = 24.91, *SD* = 6.76, *Mdn* = 25.50) than women's rating (MLQ-FF: *M* = 37.50, *SD* = 11.36, *Mdn* = 38.00; MLQ-SF: *M* = 23.57, *SD* = 7.41, *Mdn* = 24.00). Inspirational goal-oriented and passive-avoidant leadership in both MLQ forms were however not significantly different between women and men (see Supplementary Table 5 in the Supplementary Material). All leadership factors of both forms significantly but weakly correlated with age (see Table 4).

### Convergent and Predictive Validity of the MLQ-FF and the MLQ-SF - Subsample 3

In this study we examined convergent and predictive validity in subsample 3 (*n* = 691). Harman's single-factor test using principal component EFA with Kaiser criterion was employed to test for possible common-method bias. The analysis resulted in 12 distinct factors accounting for 65% of the total variance. The first unrotated factor captured 32% of the variance in the data, however no single factor emerged, and the first factor did not explain most of the variance. Thus, we assumed that CMV was not an issue in this study (Podsakoff et al., 2003).

Convergent validity was examined in the context of authentic leadership. As shown in Table 4, transformational-supportive and inspirational goal-oriented scales were positively correlated with all authentic leadership dimensions at moderate to high level. The passive-avoidant scale showed moderate to low negative correlation with authentic leadership dimensions. We thus consider the convergent validity of the tested MLQ-FF and MLQ-SF as initially supported.

The MLQ-FF and MLQ-SF factors correlated with employee's outcomes, such as work engagement, organizational commitment, work effectiveness, and satisfaction with work (Table 4). Work engagement dimensions and the total score were positively and moderately related to transformational-supportive and inspirational goal-oriented factors but very weakly to passive-avoidant factor. In addition, transformational-supportive and inspirational goal-oriented were significantly correlated with employee's organizational commitment. In general, the aggregated organizational commitment score was moderately related to transformational-supportive and inspirational goal-oriented factors but non-significantly to passive-avoidant factor. Specifically, transformational-supportive leadership and inspirational goal-oriented were moderately to highly associated with affective commitment, moderately with normative commitment, but very weakly with continuance commitment. Passive avoidant leadership correlated slightly with low employee's affective commitment and with high continuance commitment, whereas it did not significantly correlate with normative commitment. Moreover, self-perceived work effectiveness correlated weakly with high transformational-supportive and inspirational goal-oriented factors and low passive avoidant leadership. Employee's work satisfaction correlated moderately with high transformational-supportive factor, but weakly with high inspirational goal-oriented, and high passive avoidant leadership.

#### Follow-Up Study and Test-Retest Reliability

A follow-up study was conducted to assess the temporal stability of the Polish version of the MLQ. One hundred sixty-five employees (85 women, 80 men) were recruited from the total sample to complete the MLQ once again after an 8-week time interval. Participants were between 19 and 70 years old (*M* = 33.2 years; *SD* = 13.8), with mean job tenure of 10.9 years (*SD* = 12.2). The correlations between measures in two-time points were from 0.81 to 0.74 for the MLQ-FF and from 0.80 to 0.73 for the MLQ-SF (see Supplementary Table 4 in the Supplementary Material).

## Discussion

The first aim of this study was to validate the MLQ (5X Short) in the Polish organizational context. We assembled a Polish version of the transformational-transactional leadership measure called the MLQ full form or the MLQ-FF. Based on the qualitative (i.e., content analysis) and quantitative criteria (psychometric parameters) of the MLQ-FF, we then aimed at constructing a short version of the measure - the MLQ-SF. As a result, we uncovered a two- and three-factor solution of the MLQ. The two-factor solution aligns with earlier findings of active and passive leadership models (Den Hartog et al., 1997). However, we decided not to pursue this solution for the two factors did not adequately reflect the complex nature of the FRL model.

Therefore, we considered a three-factor structure of the MLQ-FF and MLQ-SF as more adequately representing specific leadership view among Polish employees. The first factor, transformational-supportive leadership, consisted of individualized consideration, attributed idealized influence, and contingent reward components, all of which reflected transformational and supportive attitudes toward employees and their work. Leaders' behaviors included approaching individuals with genuine interest, recognizing each employee's uniqueness, strength, and need for growth, creating a climate of support, encouraging innovative solutions, and facilitating employee development. The second factor, inspirational goal-oriented, was more heterogeneous and connected inspirational motivation with concern for task accomplishment and reward-for-performance exchange behaviors. Flagship behaviors of inspirational goal-oriented leadership express expectations of commitment to goals, monitoring deviances from standards, inspiring communication style, enthusiasm, and optimism. The transformational leadership items present in this factor attenuated the transactional nature of the remaining items, all of which together created a honed leadership factor of inspirational and optimistic support for the effortful achievement of organizational goals. Overall, this factor reflected behaviors such as setting direction and stimulating intrinsic employee motivation. The third factor, passive-avoidant leadership, was characterized by passive elements of the transactional component, which pertained to delaying reactions unless problems occurred, and laissez-faire behaviors.

The current three-factor structure is distinct from previously proposed three-factor models (Den Hartog et al., 1997; Avolio et al., 1999; Heinitz et al., 2005; Kanste et al., 2007; Edwards et al., 2012) in terms of factor content, explained variance, and interpretability. In previous studies, the first factor was most often represented by transformational and CR subscales (e.g., Den Hartog et al., 1997) due to their high intercorrelation (Hoogeboom and Wilderom, 2019). The second factor usually included either MBEA or MBEP items, or the mixture of both (e.g., Kanste et al., 2007; Edwards et al., 2012). In our solution, the transformational, CR, MBEA and MBEP subscales are distributed between the first two factors. In effect these factors share the meaning (Batista-Foguet et al., 2021) and are highly intercorrelated (cf. Bycio et al., 1995; Den Hartog et al., 1997; Kanste et al., 2007). Undoubtedly, this result requires further investigation, including refinement of items and scales. Alternatively, the passive-avoidant factor reflected reactive and avoiding leadership behaviors (Kanste et al., 2007; Edwards et al., 2012).

The uncovered three-factor structure of the MLQ-FF was the starting point for shortening the scale. Based on qualitative (i.e., item content analysis) and quantitative criteria (psychometric parameters), we have successfully assembled a shorter, 18-item form of the measure. The MLQ-SF included items representing all nine dimensions of the original FRL model. In fact, the novelty of the MLQ-SF lies in that it contains items from each of the nine original subscales of the MLQ-5X, thus the loss of information is minimal when compared to the full-length scale.

Both, the MLQ-FF and MLQ-SF, demonstrated good psychometric properties overall. The scale-level reliability (i.e., internal consistency and temporal stability) displayed acceptable to excellent values and were comparable with those found in previous studies that employed the MLQ (5X Short). Additionally, item-level reliability indices (i.e., item-total, inter-item, and intraclass correlations) indicated an acceptable level of discriminating power for the MLQ-FF and MLQ-SF. In sum, only a few earlier studies (cf. Tejeda et al., 2001; Heinitz et al., 2005; Kanste et al., 2007) proposed shorter versions of the MLQ (5X Short). They varied among each other across the number of factors and items considered in the measures. To our knowledge, none of these propositions have been subjected to further verification and validation.

The proposed three-factor solution shall be interpreted bearing in mind Polish contextual dynamics. Polish employees might not be cognizant of highly differentiated leadership. They may discern primarily between broad categories of leadership (Bajcar et al., 2015; Babiak et al., 2017). This mode of perception might facilitate better comprehension of the relevant leader behaviors. Also, it may help them fulfill required tasks and goals when influenced by an inspirational goal-oriented leadership or individually encouraging behaviors reflected in transformational-supportive style. Discriminating between nuanced dimensions of the MLQ (5X Short) scale may be a differentiating factor between individual employees.

Additionally, our results are inconclusive concerning differences in leadership perception in the dependence of age and gender. Undoubtfully, more research is required to test the differences in leadership perception between age groups.

The convergent and predictive validity for both, the MLQ-FF and MLQ-SF were supported. As predicted, three factors were significantly associated with authentic leadership, thus initially confirming convergent validity. According to Bass and Bass (2008), transformational and transactional leadership components indicate authenticity and ethics in behavior, consistency, transparency of values, and moral character. These virtues fall into the domain of authentic leadership, which reflects truthfulness, trustworthiness, morality, and employee-focused behavior. The passive-avoidant factor was negatively related to authentic leadership dimensions at the moderate level. Our results align with earlier findings related to associations between transformational-transactional leadership and authentic leadership (Bass and Steidlmeier, 1999; Avolio and Gardner, 2005; Lee et al., 2020).

In support of the predictive validity, MLQ-FF and the MLQ-SF factors correlated with employee's work outcomes, such as work engagement, organizational commitment, work effectiveness, and work satisfaction. Essentially, we have found positive associations of transformational-supportive and inspirational goal-oriented leadership and negative associations of passive avoidant leadership with work outcomes. The pattern of relationships between the three-factor MLQ and employee's work outcomes corresponds to the results of previous validation studies that included various measures of performance (Wang et al., 2011; Hetland et al., 2018; Steinmann et al., 2018; Lai et al., 2020), work engagement (Tims et al., 2011; Miao et al., 2012), work satisfaction (Bass et al., 2003; Sayadi, 2016; Nohe and Hertel, 2017; Kammerhoff et al., 2019), work motivation (Kanat-Maymon et al., 2020), and organizational commitment (Keegan and Hartog, 2004; Cho et al., 2019). Positively related outcomes may be thought of as psychological benefits that influence employees' overall well-being and work attitudes (Djourova et al., 2020). A rather unusual relationship was revealed between MLQ-FF and MLQ-SF factors and organizational commitment dimensions. Mainly, there are unexpected results that pertain to a very weak positive relationship between the continuance commitment dimension and passive-avoidant leadership. However, these associations are low and require further research. Thus, our findings can be considered as an initial support for the predictive validity of the three-factor MLQ-FF and MLQ-SF in Poland.

There were several limitations associated with this study. Common method variance (CMV; Podsakoff et al., 2003) may have obscured the results due to the cross-sectional design of the study. To reduce the possibility of the CMV, we have measured the variables in two-time points. Harman's single-factor test results (Podsakoff et al., 2003) indicated that CMV had not contaminated the study results. We collected data from a heterogeneous sample that was too small to scrutinize contextual aspects, such as industry, business operations, hierarchical level, age, gender, and tenure. Therefore, future research should be conducted using a larger sample of employees to explore unique leadership contexts. Moreover, the convergent validity of the MLQ-FF and the MLQ-SF need to be verified with other leadership concepts (e.g., servant, ethical, or shared leadership) and respective measures. Similarly, future studies should test the discriminant validity of the Polish version of the MLQ.

The Polish validation of the MLQ (5X Short) has theoretical and practical implications. First, the MLQ-FF and MLQ-SF enable an assessment of transformational-transactional leadership in the Polish research and organizational contexts. Scholars can potentially use both the MLQ-FF and MLQ-SF to measure employee's perception of leadership, individual and organizational antecedents, and consequences of transformational and transactional behaviors, which have not yet been investigated in the Polish-speaking population. Furthermore, the MLQ-SF, due to the ease of application and use, may serve the organizational context in the initial selection and evaluation of leaders. Preliminary screening provides a general view of a candidate's leadership potential. The mindset leaders adopt at the start of their careers can have a transformative effect on co-workers and employees. The organizational environment is highly diverse, complex, and subject to constant change. A reliable, valid, and adapted leadership style instrument is needed for practitioners and organizations concerned with effective leadership, a collaborative environment, and committed employees as fundamental elements of sustainable organizational success. Besides, the MLQ-FF may be useful when a more accurate measurement of leadership is needed. In contrast, the MLQ-SF is adequate in time-restricted leader evaluation both in the research area (like large-scale studies) and in the organizational practice (e.g., in the initial preselection of employees).

## Conclusions

This study provides academics and practitioners with a reliable, valid, and refined instrument, potentially suitable to measure Polish leaders' transformational-transactional behaviors. To our knowledge, this is the first validation study conducted among Polish employees. Along with the MLQ-FF, a shortened, 18-item form - the MLQ-SF - was proposed. We consider our research an essential part of the continuous effort to unravel the complexity of leadership and emphasize its relevance in enhancing leader-member relations - a vital force in achieving organizational performance.

## Data Availability Statement

The raw data supporting the conclusions of this article will be made available by the authors, without undue reservation.

## Ethics Statement

The studies involving human participants were reviewed and approved by the Research Ethics Committee at the Wrocław University of Science and Technology. The patients/participants provided their written informed consent to participate in this study.

## Author Contributions

Both authors contributed to the study conception, design, material preparation, and data collection. Analyses were performed by BB. In the first draft of the manuscript, introduction, and discussion was written by JB, and methods and results by BB. Both authors were involved in the discussion and revision of the final manuscript and approved the submitted version.

## Conflict of Interest

The authors declare that the research was conducted in the absence of any commercial or financial relationships that could be construed as a potential conflict of interest.

## Publisher's Note

All claims expressed in this article are solely those of the authors and do not necessarily represent those of their affiliated organizations, or those of the publisher, the editors and the reviewers. Any product that may be evaluated in this article, or claim that may be made by its manufacturer, is not guaranteed or endorsed by the publisher.

## Footnotes

^1^The presented Polish version of the ALQ was used with the permission of the Publisher of the measure (Mind Garden Inc.) and is available from the Publisher.
