# Supplementary material for: Transformational and Transactional Leadership in the Polish Organizational Context: Validation of the Full and Short Forms of the Multifactor Leadership Questionnaire
Source: Front Psychol. 2022 May 12;13:908594. doi: 10.3389/fpsyg.2022.908594 (PMC9133925; doi:10.3389/fpsyg.2022.908594)
Supplement: Supplementary file 1 [file Data_Sheet_1.docx]

Supplementary Material

**Table S1**

*Fit indexes for the MLQ (5X Short) factor models tested in the total sample*

|  | χ^2^ | df | χ^2^/df | RMSEA | SRMS | GFI | AGFI | CFI | TLI |
| --- | --- | --- | --- | --- | --- | --- | --- | --- | --- |
| 1-factor model | 2054.20 | 532 | 3.86 | .073 | .095 | .79 | .73 | .89 | .87 |
| 2-factor model v1 | 1734.04 | 531 | 3.27 | .065 | .144 | .85 | .81 | .91 | .90 |
| 2-factor model v2 | 1483.88 | 531 | 2.80 | .058 | .090 | .86 | .83 | .93 | .92 |
| 3-factor model v1 | 1483.76 | 530 | 2.80 | .058 | .092 | .86 | .83 | .93 | .91 |
| 3-factor model v2 | 1478.58 | 530 | 2.79 | .058 | .090 | .86 | .83 | .93 | .92 |
| 3-factor model v3 | 1697.46 | 530 | 3.20 | .064 | .084 | .84 | .80 | .91 | .90 |
| 3-factor model v4 | 1478.15 | 529 | 2.79 | .058 | .090 | .86 | .83 | .93 | .92 |
| 4-factor model v1 | 1475.96 | 527 | 2.80 | .058 | .090 | .86 | .83 | .93 | .92 |
| 4-factor model v2 | 1496.05 | 528 | 2.83 | .058 | .092 | .86 | .83 | .93 | .92 |
| 5-factor model | 1534.56 | 528 | 2.91 | .060 | .087 | .85 | .81 | .93 | .91 |
| 6-factor model | 1611.97 | 524 | 3.08 | .062 | .121 | .85 | .81 | .92 | .90 |
| 7-factor model | 1650.63 | 521 | 3.17 | .063 | .121 | .85 | .80 | .92 | .90 |
| 8-factor model v1 | 1718.59 | 521 | 3.30 | .065 | 133 | .84 | .80 | .91 | .89 |
| 8-factor model v2 | 2355.69 | 520 | 4.53 | .081 | .202 | .81 | .76 | .87 | .84 |
| 9-factor model | 2108.06 | 519 | 4.06 | .075 | .181 | .83 | .78 | .88 | .86 |

*Note. N* = 1065.

**Table S2**

*Factor loadings for the three-factor solution of the MLQ in subsample 1 (n = 539)*

| Item | MLQ (5X Short) subscales | | Factor 1 | Factor 2 | Factor 3 |
| --- | --- | --- | --- | --- | --- |
| MLQ19 | | IC | **.86** | -.07 | .12 |
| MLQ18 | | IIA | **.83** | .00 | .02 |
| MLQ31 | | IC | **.82** | .09 | -.02 |
| MLQ29 | | IC | **.80** | -.14 | .38 |
| MLQ30 | | IS | **.71** | .20 | .06 |
| MLQ21 | | IIA | **.71** | .13 | -.12 |
| MLQ1 | | CR | **.70** | .14 | -.12 |
| MLQ10 | | IIA | **.68** | .20 | -.05 |
| MLQ32 | | IS | **.63** | .28 | -.01 |
| MLQ23 | | IIB | **.60** | .28 | .03 |
| MLQ2 | | IS | **.60** | .24 | -.10 |
| MLQ15 | | IC | **.56** | .30 | -.01 |
| MLQ35 | | CR | **.52** | .30 | -.12 |
| MLQ13 | | IM | .02 | **.80** | .01 |
| MLQ14 | | IIB | .10 | **.74** | -.04 |
| MLQ27 | | MBEA | -.06 | **.67** | .27 |
| MLQ4 | | MBEA | -.38 | **.61** | .38 |
| MLQ26 | | IM | .22 | **.61** | -.01 |
| MLQ24 | | MBEA | .19 | **.57** | .04 |
| MLQ34 | | IIB | .27 | **.56** | .05 |
| MLQ36 | | IM | -.03 | **.56** | -.14 |
| MLQ11 | | CR | .36 | **.52** | -.08 |
| MLQ6 | | IIB | .23 | **.51** | .16 |
| MLQ9 | | IM | .30 | **.49** | -.07 |
| MLQ22 | | MBEA | .33 | **.44** | .17 |
| MLQ16 | | CR | .44 | **.44** | -.07 |
| MLQ8 | | IS | .36 | **.41** | -.19 |
| MLQ20 | | MBEP | .39 | -.14 | **.85** |
| MLQ12 | | MBEP | .15 | .05 | **.79** |
| MLQ28 | | LF | .13 | -.19 | **.77** |
| MLQ5 | | LF | -.04 | -.08 | **.76** |
| MLQ33 | | LF | -.19 | .07 | **.72** |
| MLQ7 | | LF | -.15 | -.06 | **.70** |
| MLQ17 | | MBEP | .51 | -.02 | **.52** |
| MLQ25 | | IIA | -.48 | .44 | **.52** |
| MLQ3 | | MBEP | .08 | .15 | **.45** |

*Note.* IIA – Idealized influence attributed. IIB - Idealized influence behavior. IM - Inspirational
motivation. IS - Intellectual stimulation. IC - Individualized consideration. CR – Contingent reward.
MBEA - Active management-by-exception. MBEP - Passive management-by-exception. LF - Laissez-faire.

**Table S3**

*Standardized factor loadings for MLQ-FF and MLQ-SF in the subsample 2 (n = 526)*

|  |  | Factor 1 |  | Factor 2 |  | Factor 3 |  |
| --- | --- | --- | --- | --- | --- | --- | --- |
|  |  | MLQ -FF | MLQ -SF | MLQ -FF | MLQ -SF | MLQ -FF | MLQ -SF |
| Item 21 | IIA | .75 | .75 |  |  |  |  |
| Item 18 | IIA | .72 | .72 |  |  |  |  |
| Item 30 | IS | .72 | .71 |  |  |  |  |
| Item 32 | IS | .72 | .72 |  |  |  |  |
| Item 31 | IC | .83 | .82 |  |  |  |  |
| Item 19 | IC | .52 | .54 |  |  |  |  |
| Item 1 | CR | .80 | .80 |  |  |  |  |
| Item 35 | CR | .71 | .72 |  |  |  |  |
| Item 2 | IS | .71 |  |  |  |  |  |
| Item 10 | IIA | .71 |  |  |  |  |  |
| Item 23 | IIB | .69 |  |  |  |  |  |
| Item 15 | IC | .69 |  |  |  |  |  |
| Item 29 | IC | .39 |  |  |  |  |  |
| Item 26 | IM |  |  | .71 | .72 |  |  |
| Item 13 | IM |  |  | .68 | .66 |  |  |
| Item 14 | IIB |  |  | .69 | .66 |  |  |
| Item 34 | IIB |  |  | .67 | .67 |  |  |
| Item 24 | MBEA |  |  | .57 | .60 |  |  |
| Item 27 | MBEA |  |  | .51 | .51 |  |  |
| Item 4 | MBEA |  |  | .14 |  |  |  |
| Item 36 | IM |  |  | .66 |  |  |  |
| Item 11 | CR |  |  | .73 |  |  |  |
| Item 6 | IIB |  |  | .48 |  |  |  |
| Item 9 | IM |  |  | .65 |  |  |  |
| Item 22 | MBEA |  |  | .53 |  |  |  |
| Item 16 | CR |  |  | .73 |  |  |  |
| Item 8 | IS |  |  | .72 |  |  |  |
| Item 5 | MBEP |  |  |  |  | .74 | .72 |
| Item 28 | MBEP |  |  |  |  | .75 | .73 |
| Item 20 | LF |  |  |  |  | .75 | .78 |
| Item 12 | LF |  |  |  |  | .66 | .73 |
| Item 3 | MBEP |  |  |  |  | .33 |  |
| Item 7 | LF |  |  |  |  | .70 |  |
| Item 17 | LF |  |  |  |  | .17 |  |
| Item 33 | MBEP |  |  |  |  | .72 |  |

*Note.* MLQ-FF: full form of the MLQ. MLQ-SF: short form of the MLQ. *Note.* IIA – Idealized influence attributed.
IIB - Idealized influence behavior. IM - Inspirational motivation. IS - Intellectual stimulation. IC - Individualized consideration.
CR – Contingent reward. MBEA - Active management-by-exception. MBEP - Passive management-by-exception. LF - Laissez-faire.

**Table S4**

*Discriminating power and reliability coefficients for the three MLQ-FF and MLQ-SF factors*

| MLQ Factor | F1: Transformational-supportive | | F2: Inspirational  goal-oriented | | F3: Passive -avoidant | |
| --- | --- | --- | --- | --- | --- | --- |
| MLQ form | MLQ-FF | MLQ-SF | MLQ-FF | MLQ-SF | MLQ-FF | MLQ-SF |
| Number of items | 13 | 8 | 14 | 6 | 8 | 4 |
| *Subsample 1 (n = 539)* |  |  |  |  |  |  |
| Cronbach α | .96 | .94 | .91 | .86 | .84 | .80 |
| ICC^a^ | .95 | .94 | .91 | .86 | .84 | .80 |
| Inter-item mean correlation | .62 | .66 | .43 | .51 | .39 | .51 |
| Inter-item correlations | .36 - .82 | .54 - .79 | .01 - .75 | .34- .75 | .09 - .68 | .44 - .62 |
| Item-total correlations | .50 - .87 | .75 - .86 | .14 - .77 | .47 - .73 | .28 - .68 | .60 - .63 |
| *Subsample 2 (n = 526)* |  |  |  |  |  |  |
| Cronbach α | .93 | .91 | .90 | .82 | .83 | .83 |
| CR | .92 | .90 | .89 | .80 | .83 | .83 |
| AVE | .49 | .53 | .39 | .41 | .41 | .55 |
| ICC^a^ | .93 | .90 | .90 | .82 | .83 | .83 |
| Inter-item mean correlation | .49 | .54 | .39 | .43 | .38 | .55 |
| Inter-item correlations | .21 - .76 | .38 - .76 | .10 - .67 | .28 - .60 | .05 - .59 | .51 - .59 |
| Item-total correlations | .41 - .82 | .52 - .80 | .27 - .69 | .48 - .65 | .19 - .70 | .63 - .70 |
| r_tt_^2b^ | .79^**^ | .80^**^ | .81^**^ | .76^**^ | .73^**^ | .73^**^ |

*Note.* ^a^ ICC – Intra-class correlation. ^b^ *r_tt_* – test-retest correlation (*n* = 165). *^**^ p <* .01. MLQ-FF: full form of the MLQ.
MLQ-SF: short form of the MLQ.

**Table S5**

*Gender differences in MLQ-FF and MLQ-SF factors in subsamples 1 and 2*

|  | Women | | | Men | | | *U* | *z* | *p* |
| --- | --- | --- | --- | --- | --- | --- | --- | --- | --- |
|  | *M* | *SD* | *Mdn* | *M* | *SD* | *Mdn* |  |  |  |
| *Subsample 1^a^ (n = 539)* |  |  |  |  |  |  |  |  |  |
| MLQ-FF |  |  |  |  |  |  |  |  |  |
| 1. Transformational-supportive | 37.79 | 12.16 | 39.00 | 37.97 | 10.74 | 39.00 | 35931.00 | -.05 | .963 |
| 2. Inspirational goal-oriented | 43.56 | 9.86 | 43.00 | 43.39 | 9.32 | 43.00 | 35979.50 | -.02 | .984 |
| 3. Passive-avoidant | 21.32 | 5.97 | 21.00 | 22.35 | 5.52 | 22.00 | 31633.50^*^ | -2.44 | .015 |
| MLQ-SF |  |  |  |  |  |  |  |  |  |
| 1. Transformational-supportive | 23.55 | 7.86 | 24.00 | 23.69 | 6.89 | 24.00 | 35787.00 | -.13 | .899 |
| 2. Inspirational goal-oriented | 18.47 | 4.75 | 18.00 | 18.23 | 4.42 | 18.00 | 35109.00 | -.505 | .613 |
| 3. Passive-avoidant | 10.14 | 3.41 | 10.00 | 10.62 | 3.19 | 11.00 | 32375.00^*^ | -2.03 | .042 |
|  |  |  |  |  |  |  |  |  |  |
| *Subsample 2^b^ (n = 526)* |  |  |  |  |  |  |  |  |  |
| MLQ-FF |  |  |  |  |  |  |  |  |  |
| 1. Transformational-supportive | 37.50 | 11.36 | 38.00 | 39.65 | 10.22 | 41.00 | 30376.50^*^ | -2.35 | .019 |
| 2. Inspirational goal-oriented | 43.72 | 10.35 | 44.00 | 44.35 | 9.38 | 44.50 | 33513.00 | -.55 | .581 |
| 3. Passive-avoidant | 19.78 | 5.96 | 19.00 | 20.16 | 6.02 | 20.00 | 32923.00 | -.89 | .373 |
| MLQ-SF |  |  |  |  |  |  |  |  |  |
| 1. Transformational-supportive | 23.57 | 7.41 | 24.00 | 24.91 | 6.76 | 25.50 | 30563.00^*^ | -2.25 | .025 |
| 2. Inspirational goal-oriented | 18.55 | 4.79 | 19.00 | 18.95 | 4.52 | 19.00 | 32682.00 | -1.03 | .302 |
| 3. Passive-avoidant | 9.21 | 3.61 | 9.00 | 9.49 | 3.60 | 9.00 | 32816.00 | -.95 | .339 |

*Note.* U – Mann-Whitney test statistic. z – standardized test statistic. ^a^ subsample 1: women *n =* 294, men *n =* 245. ^b^subsample 2: women *n =* 278,
men *n =* 248. MLQ-FF: full form of the MLQ. MLQ-SF: short form of the MLQ.
